# Experimental dataset of masonry prisms with hollow concrete blocks

**DOI:** 10.1016/j.dib.2024.110207

**Published:** 2024-02-19

**Authors:** Jorge H. Chávez-Gómez, José Álvarez-Pérez, Milena Mesa-Lavista, Ramón García-Cedeño, Franco A. Carpio-Santamaría, G. Fajardo-San-Miguel, Fabiola D. Yépez-Rincón

**Affiliations:** a Universidad Autónoma de Nuevo León (UANL), Facultad de Ingeniería Civil (FIC), Departamento de Estructuras, Av. Universidad, s/n CP. 66455, San Nicolás de los Garza, Nuevo León, México; b Universidad Veracruzana (UV), Instituto de Ingeniería, S. S. Juan Pablo II, Zona Universitaria, Boca del Río 94294, México

**Keywords:** Compressive strength, Prisms, Full-shell mortar placement, Face-shell bedding mortar placement

## Abstract

The uniaxial compressive strength is a main parameter to be considered in design of masonry structures, particularly due to gravitational loads. This dataset collection comprises test on various masonry prisms made of hollow concrete blocks. These prisms are divided into three groups: prisms consisting of 2, 3, and 4 courses, respectively. All prisms underwent testing with mortar applied over the net area (full-shell) and on the lateral face (face-shell-bedding). Ten prisms were tested for each arrangement, meaning 10 prisms with 2 courses using full-shell placement, 10 with face-shell placement, and so forth for 3 and 4 course prisms. Two linear variable differential transformers (LVDTs) were added to every prism for displacement measurements. Load measurements were conducted by using an Instron machine for the prisms with two courses, a Tinius Olsen machine for those with three courses, and a loading frame for the four-course prisms. The presented dataset includes strain-stress curves that underwent analysis and filtering. These data hold significance as numerous researchers conduct projects in this domain, and experimental results play a crucial role in validating numerical and analytical models.

Specifications Table

**Table:** 

Subject	Civil and Structural Engineering
Specific subject area	Strength compression in masonry of hollow concrete blocks
Data format	Analysed and Filtered
Type of data	Tables and Images in excel sheets Folder with photos (numbered by test)
Data collection	The Universal Testing Machine Instron, equipped with a 60-tons load capacity, conducted tests on prisms comprising two courses under a displacement control. For the three-course prisms, a servo-hydraulic Tinius Olsen machine, with a load capacity of 200 tons, was employed, also by using displacement control. To test the four-course prisms, a custom loading frame was developed, operating under load control. This setup included a load-cell capacity of 100 tons and a hydraulic jack capacity of 60 tons. Additionally, two LVDTs were installed in each prism to precisely measure displacement.
Data source location	Institution: Universidad Autónoma de Nuevo León, Instituto de Ingeniería Civil. City: San Nicolás de los Garza Town: Ciudad Universitaria Region: Nuevo León Country: Mexico (25°44’00.07’’ N, 100°18’22.55’’ W)
Data accessibility	Repository name: Mendely data Data identification number: 10.17632/9bmhxgdsk2.2 Direct URL to data: https://data.mendeley.com/datasets/9bmhxgdsk2/2

## 1 Value of the Data

• The dataset showcases the results obtained from testing masonry prisms constructed with hollow concrete blocks under compressive strength. Ten prisms for each of two, three, and four courses were tested with mortar placed over the full-shell, and ten prisms for each course arrangement with mortar placed over the face-shell-bedding.
• The provided data is useful for researchers in masonry structures, particularly due to the different standards concerning prism courses and methods of mortar placement.
• These data provide valuable insights for comparative analysis with other experimental results and serve as essential inputs for finite element method modeling.
• In current research, datasets play a crucial role in the validation and calibration formulations designed for masonry structure construction. However, when it comes to experimental results concerning hollow concrete blocks in masonry, the available data are often scarce, in particular the face-shell bedding configuration. Thus, the data obtained from these experiments prove to be exceptionally valuable.
• The presented data is also valuable in professional engineering practice, as current trends lean towards construction using mortar placed in the face-shell-bedding technique. This allows for relevant comparisons to be made when using mortar in full-shell and face-shell configurations.

## 2 Background

Masonry structural design utilizing hollow concrete blocks is prevalent worldwide due to its widespread implementation. Among the main factors to consider, uniaxial compressive strength stands out prominently. A key variable that impacted recent masonry design is the mortar placement technique. Diverse design standards take varied stances on this matter. For instance, the Mexican standards [1], [2], [3] advocate for mortar placement covering the full shell (over the net area), with strength computations based on the gross area. Conversely, the United States of America standards [4,5] recommend placing mortar over the net area, aligning computation with this placement. In contrast, the Canadian standard [6] specifies mortar placement on the lateral face (face shell bedding), utilizing the effective area of load—i.e., the area of mortar placed—for computations. The Australian standard [6] has a distinct approach, differentiating between face-shell-bedding and full-shell placements. Eurocode [7] does not distinguish between face and full placements. This diversity prompted research endeavours involving prism tests under compressive strength, exploring various methods of mortar placement in masonry structures. This dataset caters to researchers seeking to delve into the influence of mortar placement on the behaviour of masonry structures.

## 3 Data Description

**Fig. 1:**
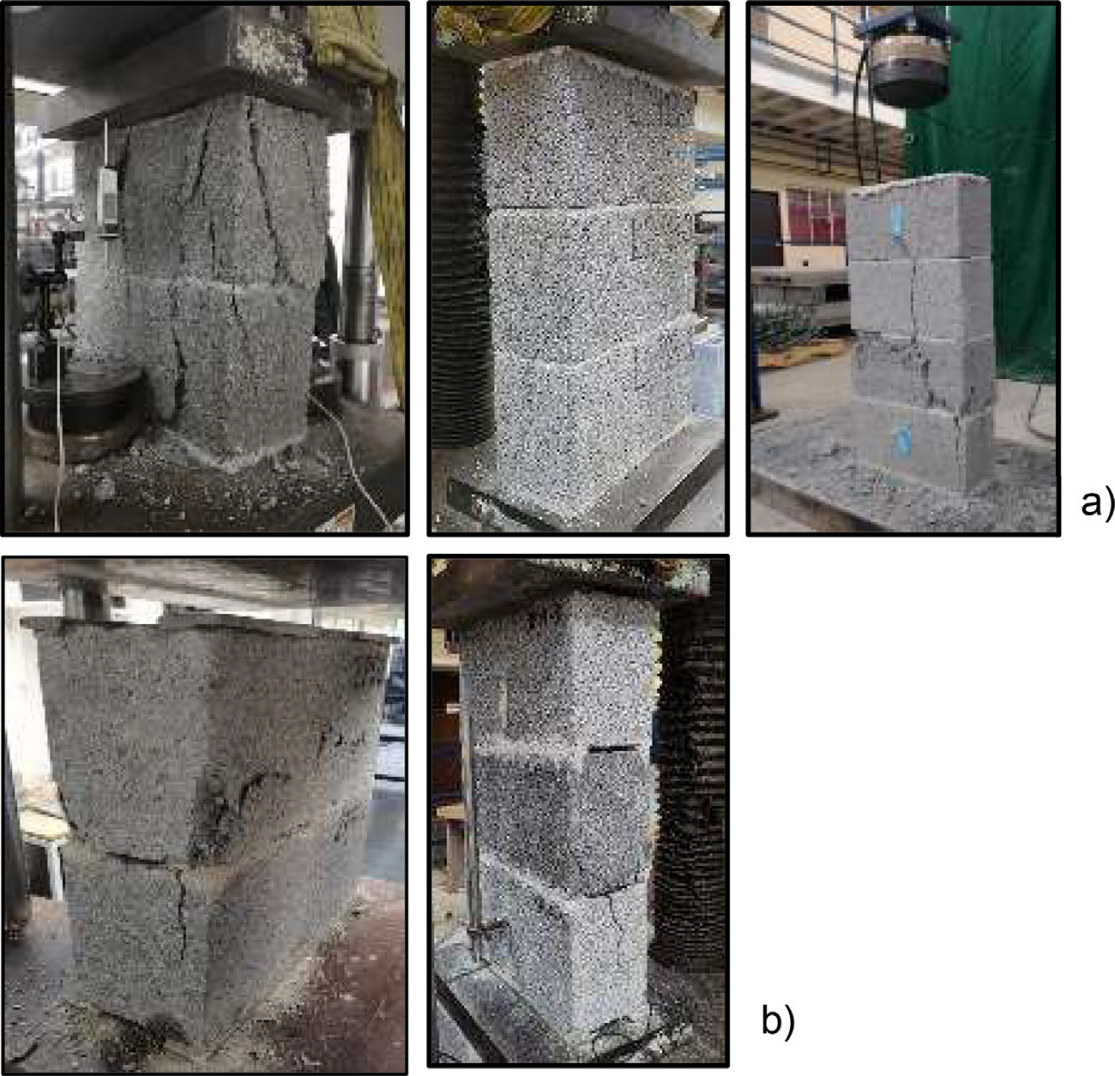
Prism of 2, 3 and 4 courses tested: a) with mortar placement over the net area (full-shell), b) with mortar placement over the lateral face (face-shell-bedding).

The dataset showcases results, from prism tests conducted under uniaxial compression. Fig. 1 illustrates the prisms tested in arrangements of 2, 3, and 4 courses, with mortar placed over the net area (Fig. 1a) and over the lateral face (Fig. 1b). The dataset comprises five files containing pre-test and post-test photos of the specimens, offering researchers a visual understanding of specimen failure. However, in instances where failures were explosive, such as with the 4-course prisms in face-shell-bedding arrangements, any photo could not be obtained.

Furthermore, the results are organized in three Excel files, each containing processed data for prisms consisting of 2, 3, and 4 courses respectively, considering both full-shell and face-shell-bedding placements.

**Fig. 2:**
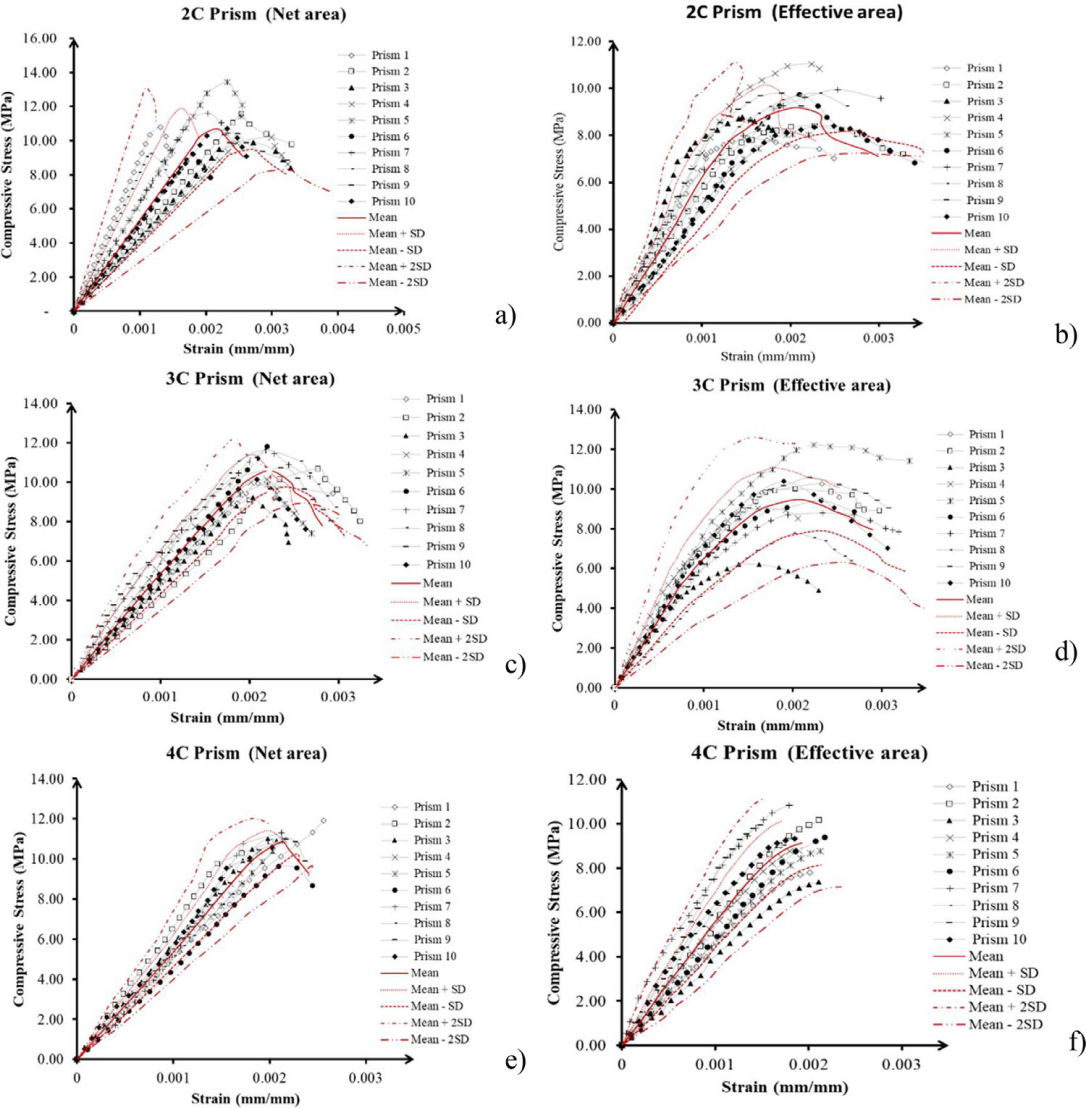
Stress – strain curves in prisms of a) two courses in full-shell over the net area, b) two courses in face-shell-bedding over effective area, c) three courses in full-shell over the net area, d) three courses in face-shell-bedding over effective area, e) four courses in full-shell over the net area, and f) four courses in face-shell-bedding over effective area.

The dataset includes stress-strain graphics (Fig. 2), displaying mean values alongside one and two standard deviations. Stress-strain curves were generated for effective areas in face-shell-bedding and gross and net areas in full-shell arrangements, aligning with varying standards' requirements.

A summary of main parameters for prisms of two, three and four courses is provided in Table 1, Table 2, Table 3 respectively. These tables encompass elastic modulus, maximum compressive strength, maximum and ultimate strain, and ductility factor. The complete dataset, along with photos can be found at https://data.mendeley.com/datasets/9bmhxgdsk2/2, as there is an extensive amount of data.

**Table 1:** Summary of calculated parameters for prisms of two courses.

Two courses with mortar placed over the net area (full-shell)					
Samples	E_cm(40%)_ (MPa)	Compressive strength (MPa)	Strain at maximum load	Ultimate Strain	Ductility Factor
Prism 1	9013	10.79	0.0013	0.0015	1.11
Prism 2	4587	11.53	0.0025	0.0033	1.30
Prism 3	4311	9.89	0.0027	0.0033	1.21
Prism 4	4010	10.20	0.0030	0.0032	1.08
Prism 5	6327	13.44	0.0023	0.0025	1.09
Prism 6	5167	9.21	0.0018	0.0021	1.15
Prism 7	6448	11.62	0.0020	0.0024	1.19
Prism 8	8224	9.24	0.0012	0.0014	1.24
Prism 9	4183	10.40	0.0025	0.0032	1.30
Prism10	5111	10.70	0.0023	0.0026	1.13
**Mean**	**5738**	**10.70**	**0.0022**	**0.0026**	**1.18**
**St. Desv.**	**1651**	**1.20**	**0.0006**	**0.0007**	**0.08**
**COV**	**0.29**	**0.11**	**0.26**	**0.27**	**0.06**

Two courses with mortar placed over face-shell					

Prism 1	6255	7.90	0.0015	0.0025	1.65
Prism 2	5651	8.41	0.0023	0.0040	1.75
Prism 3	8430	8.78	0.0015	0.0020	1.34
Prism 4	7303	11.05	0.0022	0.0023	1.04
Prism 5	4711	8.05	0.0020	0.0022	1.09
Prism 6	4807	9.74	0.0021	0.0034	1.62
Prism 7	6665	9.96	0.0025	0.0030	1.19
Prism 8	5872	9.79	0.0023	0.0026	1.13
Prism 9	7276	9.79	0.0019	0.0021	1.12
Prism10	4716	8.41	0.0025	0.0031	1.25
**Mean**	**6169**	**9.19**	**0.0021**	**0.0027**	**1.32**
**St. Desv.**	**1197**	**0.97**	**0.0004**	**0.0008**	**0.30**
**COV**	**0.19**	**0.11**	**0.17**	**0.28**	**0.23**

**Table 2:** Summary of calculated parameters for prisms of three courses.

Three courses with mortar placed over the net area (full-shell)					
Samples	E_cm(40%)_ (MPa)	Compressive strength (MPa)	Strain at maximum load	Ultimate Strain	Ductility Factor
Prism 1	4803	9.92	0.00209	0.0033	1.60
Prism 2	4152	10.68	0.00277	0.0032	1.17
Prism 3	4741	9.24	0.00203	0.0024	1.20
Prism 4	5764	10.15	0.00219	0.0024	1.11
Prism 5	4966	9.88	0.00220	0.0027	1.23
Prism 6	5356	11.79	0.00220	0.0022	1.00
Prism 7	6857	11.62	0.00218	0.0027	1.23
Prism 8	5894	11.63	0.00217	0.0030	1.37
Prism 9	7744	10.72	0.00235	0.0029	1.24
Prism10	5148	10.15	0.00208	0.0026	1.26
**Mean**	**5543**	**10.58**	**0.0022**	**0.0028**	**1.24**
**St. Desv.**	**1019**	**0.82**	**0.0002**	**0.0003**	**0.14**
**COV**	**0.18**	**0.08**	**0.09**	**0.11**	**0.11**

Three courses with mortar placed over face-shell					

Prism 1	7575	10.26	0.0023	0.0025	1.08
Prism 2	7272	10.01	0.0020	0.0030	1.47
Prism 3	7542	6.21	0.0016	0.0023	1.41
Prism 4	8913	9.02	0.0016	0.0021	1.28
Prism 5	7803	12.22	0.0022	0.0033	1.48
Prism 6	6466	9.42	0.0023	0.0027	1.16
Prism 7	6913	8.79	0.0023	0.0032	1.37
Prism 8	4744	7.77	0.0020	0.0026	1.32
Prism 9	6141	10.58	0.0022	0.0031	1.41
Prism10	7341	10.38	0.0019	0.0031	1.61
**Mean**	**7071**	**9.47**	**0.0021**	**0.0030**	**1.42**
**St. Desv.**	**1058**	**1.57**	**0.0003**	**0.0003**	**0.07**
**COV**	**0.15**	**0.17**	**0.13**	**0.09**	**0.05**

**Table 3:** Summary of calculated parameters for prisms of four courses.

Four courses with mortar placed over the net area (full-shell)					
Samples	E_cm(40%)_ (MPa)	Compressive strength (MPa)	Strain at maximum load	Ultimate Strain	Ductility Factor
Prism 1	5031	11.32	0.0024	0.0026	1.05
Prism 2	6818	10.29	0.0017	0.0020	1.17
Prism 3	5765	10.48	0.0018	0.0021	1.14
Prism 4	5490	10.39	0.0019	0.0022	1.14
Prism 5	5120	10.39	0.0020	0.0021	1.05
Prism 6	4434	9.13	0.0020	0.0024	1.25
Prism 7	4958	10.74	0.0017	0.0021	1.23
Prism 8	5091	9.77	0.0019	0.0020	1.05
Prism 9	5518	10.44	0.0019	0.0024	1.25
Prism10	5681	10.05	0.0018	0.0020	1.13
**Mean**	**5391**	**10.30**	**0.0019**	**0.0022**	**1.1461**
**St. Desv.**	**607**	**0.55**	**0.0002**	**0.0002**	**0.0520**
**COV**	**0.11**	**0.05**	**0.10**	**0.07**	**0.05**

Four courses with mortar placed over face-shell					

Prism 1	4373	7.80	0.0020	0.0020	1.00
Prism 2	5283	10.16	0.0021	0.0021	1.00
Prism 3	3734	7.37	0.0021	0.0021	1.00
Prism 4	5478	8.81	0.0019	0.0019	1.00
Prism 5	4721	8.77	0.0021	0.0021	1.00
Prism 6	4717	9.38	0.0022	0.0022	1.00
Prism 7	8447	10.84	0.0018	0.0018	1.00
Prism 8	6365	9.03	0.0017	0.0017	1.00
Prism 9	7060	9.94	0.0015	0.0015	1.00
Prism10	6724	9.32	0.0018	0.0018	1.00
**Mean**	**5690**	**9.14**	**0.0019**	**0.0019**	**1.00**
**St. Desv.**	**1366**	**0.99**	**0.0002**	**0.0002**	**-**
**COV**	**0.24**	**0.11**	**0.11**	**0.11**	**-**

## 4 Experimental Design, Materials and Methods

**Fig. 3:**
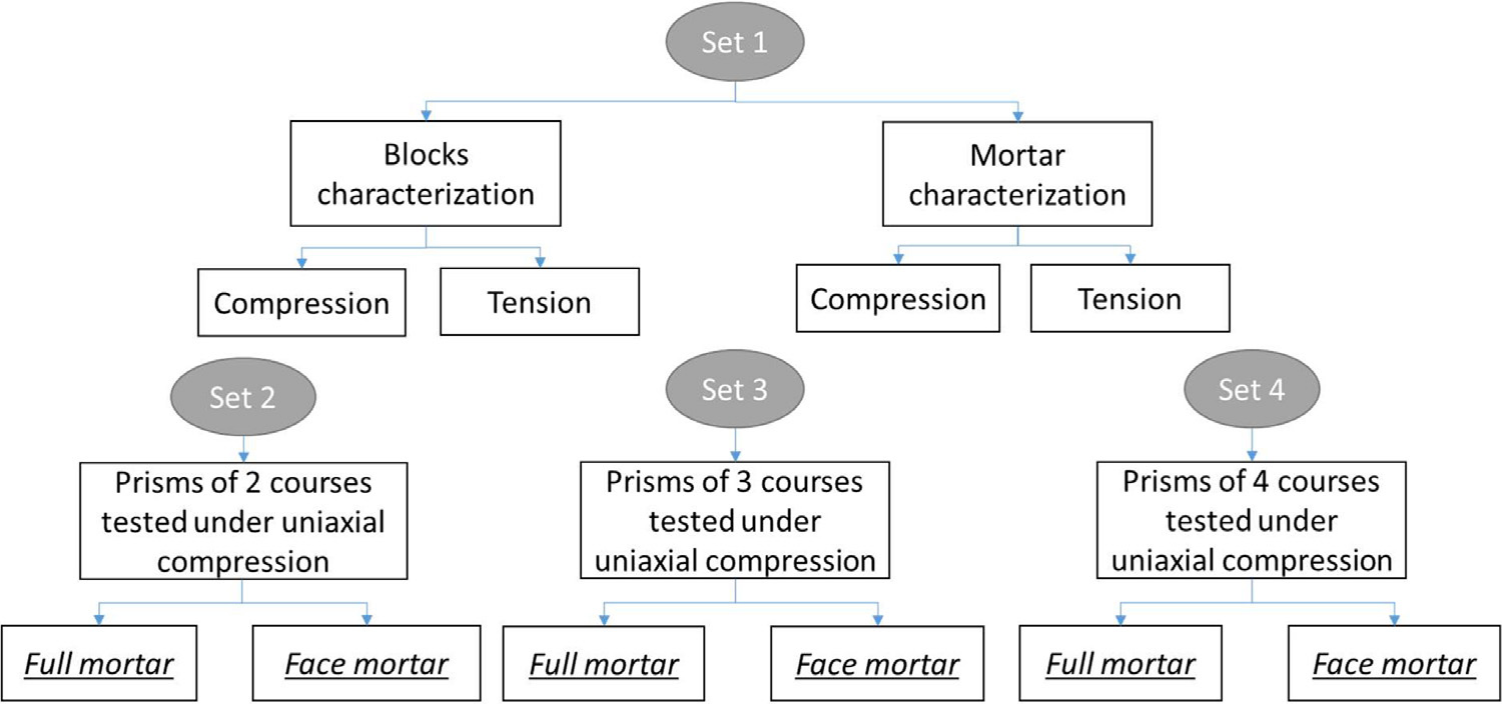
Flowchart to experimental design.

**Fig. 4:**
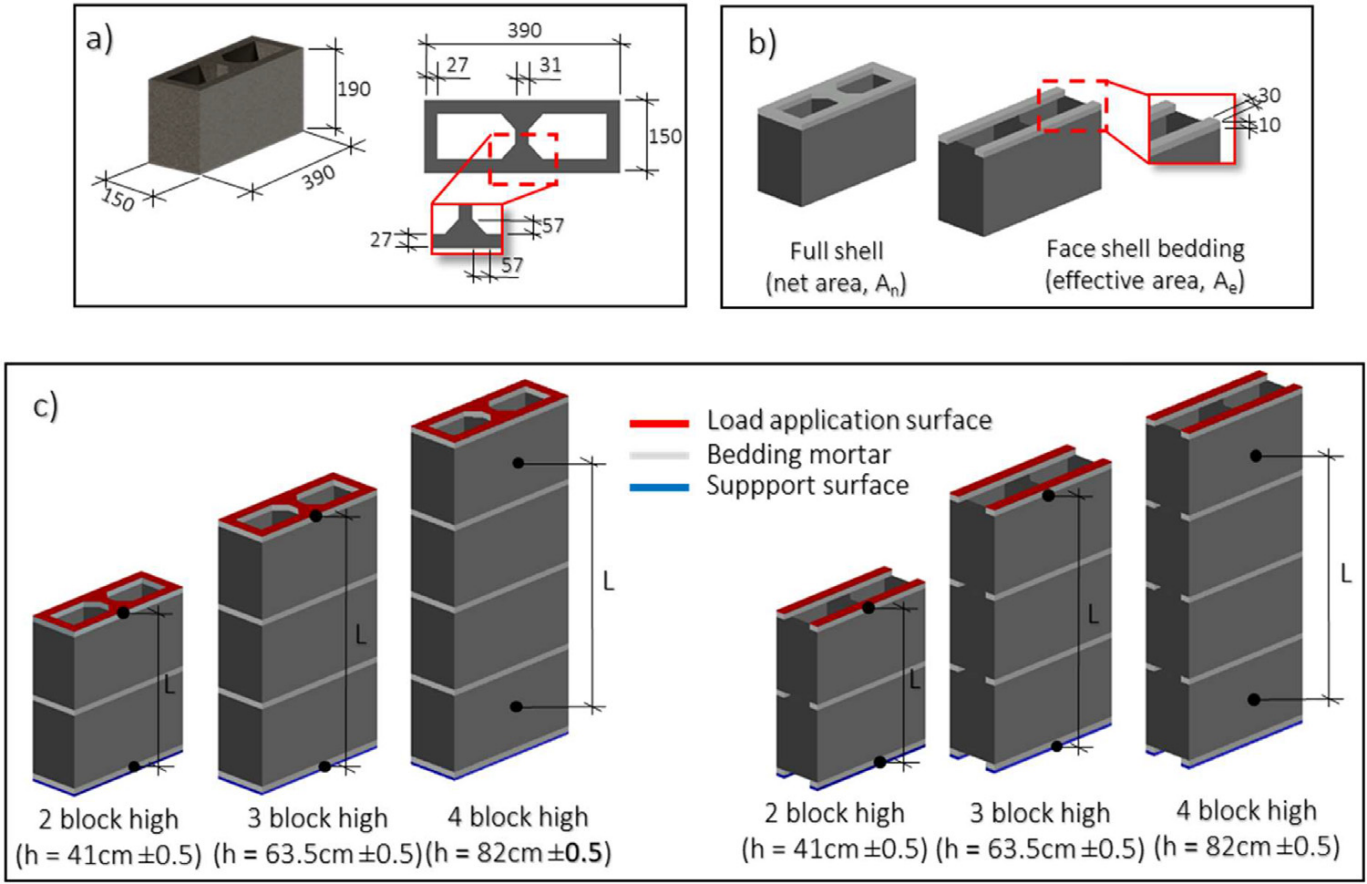
Description of tested prisms and their measurements, a) Hollow concrete block geometry (dimension in mm), b) Mortar bedding and its configuration (dimensions in mm), c) Set of prisms (dimensions in cm)

The experimental design's flowchart is presented in Fig. 3. In a set 1, the component materials of the masonry (mortar and blocks) were characterized, obtaining its physical and mechanical properties of tensile and compressive strength [3,5]. In a second stage, the masonry prisms were constructed by using hollow concrete blocks with two holes, featuring nominal dimensions of 390×190×150 mm (Fig. 4). These blocks were sourced from the same batch, with a mean block compressive strength over the net area (A_n_ =327 cm^2^) of $f{{}^{\prime }}_{C_{n}}=11.27\;\text{MPa}$ and a mean tensile strength of $f_{t}=0.90\;\text{MPa}$ (Fig. 4a).

The mortar employed in prism construction exhibited a mean compressive strength of 17.09 MPa and a mean tensile strength of 3.13 MPa, obtained from cubes (edges of 50 mm) and briquettes, respectively. The thickness of the joint was 10 mm (Fig. 4b).

Subsequently, three sets of ten prisms were built for each course configuration (2, 3, and 4 courses), with mortar placed in both full-shell and face-shell bedding arrangements (Fig. 4c). The displacements and deformations were measured, using LVDT and strain gauges. The LVDTs were placed in pairs, located one on each side in the prisms center. In the prisms of 2 and 3 courses, the instrumented length (L) was between load surface (red surface from Fig. 4c) and support surface (blue surface form Fig. 4c). On the other hand, in prisms of 4 courses, instrumented length was between bottom and upper block. After 28 days, the prisms were tested, as per the procedure of Mexican standards [1], [2], [3], load-displacement and stress-deformation curves were obtained.

Given that the data was acquired in terms of load-displacement curve, the stress and strain values were calculated. Concerning the stress values, the load was divided by the respective net (A_n_ = 327 cm^2^) and gross (A_g_ = 585 cm^2^) areas for prisms tested in full-shell configurations. Conversely, for prisms tested in face-shell bedding configurations, the load was divided by the effective area (A_e_ = 234 cm^2^) to obtain stress values. Additionally, strain values were calculated by dividing the measured displacement by the corresponding length (L) (Fig. 4c).

Table 4 provides a summary of the tested specimens, the instrumentation utilized, and the data acquisition process for each specimen. Once the measurements for load, displacement, and strain were taken, the Microsoft Office application was employed for data processing.

**Table 4:** Summary of tested specimens, instrumentations and the data acquisition.

Parameters	Prisms		
	2 courses	3 courses	4 courses
Machine	Instron	Tinius Olsen	Load frame
Capacity	60 tons	200 tons	100 tons
Limitations	Testing machine limited to specimens of 60 cm height	Testing machine limited to specimens of 90 cm height	Not allows displacement control
Data obtained	displacement control 0.005mm/sec	displacement control 0.005mm/sec	load control 37kPa/sec
Instrumentation for displacement acquisition	2 LVDTs in all specimens and the LVDT from the machine	2 LVDTs in all specimens	2 LVDTs in all specimens
Instrumentation for strain acquisition	Strain gauges in some specimens	Strain gauges in some specimens	Strain gauges in some specimens
Data acquisition	Data acquisition unit NI PXIe-1073 NI TB-4330 Bridge Input Card NI TB-4340 LVDT Card	Data acquisition unit NI PXIe-1073 NI TB-4330 Bridge Input Card NI TB-4340 LVDT Card	Data acquisition unit NI PXIe-1073 NI TB-4330 Bridge Input Card NI TB-4340 LVDT Card
Specimens	10 in full 10 in face	10 in full 10 in face	10 in full 10 in face
Mean values of specimen height	410 mm	635 mm	820 mm
Instrumentation height (spherical seat and plates)	≈20 cm	≈20 cm	≈20 cm

## Limitations

This data specifically belongs to masonry constructed with hollow concrete blocks featuring two holes and a mean compressive strength of 11.27 MPa over the net area. It is essential to note that these results should not be extrapolated to other types of blocks or construction materials.

## Ethics Statement

The authors have read and followed the ethical requirements for publication in Data in Brief and confirm that the current work does not involve human subjects, animal experiments, or any data collected from social media platforms.

## CRediT authorship contribution statement

**Jorge H. Chávez-Gómez:** Resources, Funding acquisition, Writing – review & editing. **José Álvarez-Pérez:** Supervision, Formal analysis. **Milena Mesa-Lavista:** Conceptualization, Writing – original draft. **Ramón García-Cedeño:** Data curation, Investigation, Validation. **Franco A. Carpio-Santamaría:** Visualization, Writing – review & editing. **G. Fajardo-San-Miguel:** Methodology, Writing – review & editing. **Fabiola D. Yépez-Rincón:** Investigation, Writing – review & editing.

## Data Availability

Uniaxial Compressive Strength Testing of Hollow Concrete Block Prisms (Original data) (Mendeley Data).
